# The North Italian Longitudinal Study Assessing the Mental Health Effects of SARS-CoV-2 Pandemic on Health Care Workers—Part I: Study Design and Psychometric Structural Validity of the HSE Indicator Tool and Work Satisfaction Scale

**DOI:** 10.3390/ijerph19159514

**Published:** 2022-08-03

**Authors:** Giovanni Veronesi, Emanuele Maria Giusti, Alessia D’Amato, Francesco Gianfagna, Rossana Borchini, Gianluca Castelnuovo, Licia Iacoviello, Marco Mario Ferrario

**Affiliations:** 1 EPIMED Research Center, Department of Medicine and Surgery, University of Insubria, 21100 Varese, Italy; giovanni.veronesi@uninsubria.it (G.V.); francesco.gianfagna@uninsubria.it (F.G.); licia.iacoviello@uninsubria.it (L.I.); 2 Psychology Research Laboratory, Istituto Auxologico Italiano IRCCS, 20149 Milan, Italy; e.giusti@auxologico.it; 3 Department of Psychology, Catholic University of the Sacred Heart, 20123 Milan, Italy; gianluca.castelnuovo@unicatt.it; 4 ASST Sette Laghi, 21100 Varese, Italy; alessia.damato@gmail.com; 5 Mediterranea Cardiocentro, 80122 Napoli, Italy; 6 UOS Medicina Preventiva e Legale, ASST Lariana, 22100 Como, Italy; rossana.borchini@asst-lariana.it; 7 Psychology Research Laboratory, Istituto Auxologico Italiano IRCCS, 28824 Verbania, Italy; 8 Department of Epidemiology and Prevention, IRCCS Neuromed, 86077 Pozzilli, Italy

**Keywords:** mental health, health care workers, HSE indicator tool, work satisfaction, principal component analysis, COVID-19, longitudinal study

## Abstract

Literature on the impact of the SARS-CoV-2 pandemic on the mental health of Health Care Workers (HCWs) is mostly based on cross-sectional surveys. We designed a longitudinal study to assess work-related stress and mental health before and after the pandemic onset in a university-hospital in Lombardia region, Italy. We report on sample representativeness and structural validity of questionnaires assessing work stress (HSE Indicator Tool, HSE-IT) and work satisfaction (WS), which were not validated in the HCWs population. n = 1287 HCWs from 67 hospital wards/offices were invited to an online survey in summer 2019 (pre-COVID-19 wave) and again during winter 2020 (COVID-19 wave). Selected hospital wards/offices did not differ from the remaining wards for turn-over and down-sizing rates, overload, sick leaves, and night shifts (Wilcoxon rank tests *p*-values > 0.05). Participation rates were 70% (n = 805) and 60% (n = 431) in the pre-COVID-19 and COVID-19 waves, respectively. Socio-demographic and work-related characteristics did not impact data completeness nor participation to the COVID-19 wave. While confirming a 7-component structure for HSE-IT, we identified a new factor related to participation in work organization. A one-factor model for WS had satisfactory fit. Our longitudinal study based on a representative sample and adopting validated questionnaires is well-suited to elucidate the role of work conditions on the development of mental health disorders in HCWs.

## 1. Introduction

From the inception of the COVID-19 pandemic, health care workers (HCWs) have been on the frontline against the disease, being one of the most exposed populations [1,2]. Along with the risk of contagion, the shortage of efficient protective equipment, and the moral distress due to the lack of effective treatments to fight the virus [3], HCWs were under overwhelming pressure due to institutional constraints. These include increased workload due to long working hours and irregular shifts, as well as frequent and sudden reorganizations of wards and departments occurring in a short time period to comply with the exponential growth of the pandemic. Coping with all these concurrent challenges is having severe consequences on the HCWs population in terms of wellbeing and mental health [4].

Since the onset of the pandemic, a large number of studies investigated its “impact” on burnout, post-traumatic stress disorders (PTSD), anxiety and depressive symptoms among HCWs [5,6,7,8,9,10,11,12]. Most of them were conducted using a cross-sectional design and with sampling strategies that are unable to assess and mitigate the risk of selection bias. All the 16 cross-sectional studies in the meta-analysis by Kisely et al. [5] were based on convenient samples or snowball sampling; nine did not report participation rates, and two had participation below 40%. In one study in Italy, the use of a convenient sample and the lack of psychometric proprieties in the adopted scales resulted in un-expected and counterintuitive associations between stress and burnout [12,13]. Moreover, the effect of exposures to pre-existing organizational constraints and work stress [14], as well as the pre-pandemic levels of the investigated mental health conditions on the same subjects are methodological issues of major importance to disentangle the true effect of the pandemic on mental health from that of other different components. Furthermore, the potential lack of measurement invariance of the adopted questionnaires after the pandemic outbreak needs to be assessed, as life stressors, natural disasters and major contextual changes might alter the psychometric characteristics of questionnaires [15].

The impact of pre-existing stressful working conditions on the mental health of HCWs have been widely documented [14,16,17]. Besides age and female gender, shift work scheduling, overload, poor sleep quality and circadian rhythm alteration have been associated with burnout onset [14]. Several longitudinal studies established a link between increasing work demand, decreasing control and managers’ and peers’ support with burnout incidence [16,17]. The valid assessment of these conditions in HCWs is therefore important. In Italy, the Health and Safety Executive Management Standards Indicator Tool (HSE) is the standard instrument to assess perceived work stress as recommended by the regulatory labor safety agency [18,19]. Nonetheless, studies assessing its structural validity employed heterogeneous methods and obtained different results [20,21,22]. In addition, the evaluation of structural validity of the Italian version of the HSE in HCWs is lacking.

We designed a longitudinal study with the aim to assess the mental health effects of SARS-CoV-2 pandemic on HCWs, considering the pre-pandemic levels of work strain, work satisfaction and burnout. In this paper, we report on sample representativeness and structural validity of the HSE and of a work satisfaction scale. A parallel paper will address the structural validity and the longitudinal invariance of the selected scales to appraise mental health disorders [23].

## 2. Materials and Methods

### 2.1. Study Population, Design and Recruitment

**Figure 1:**
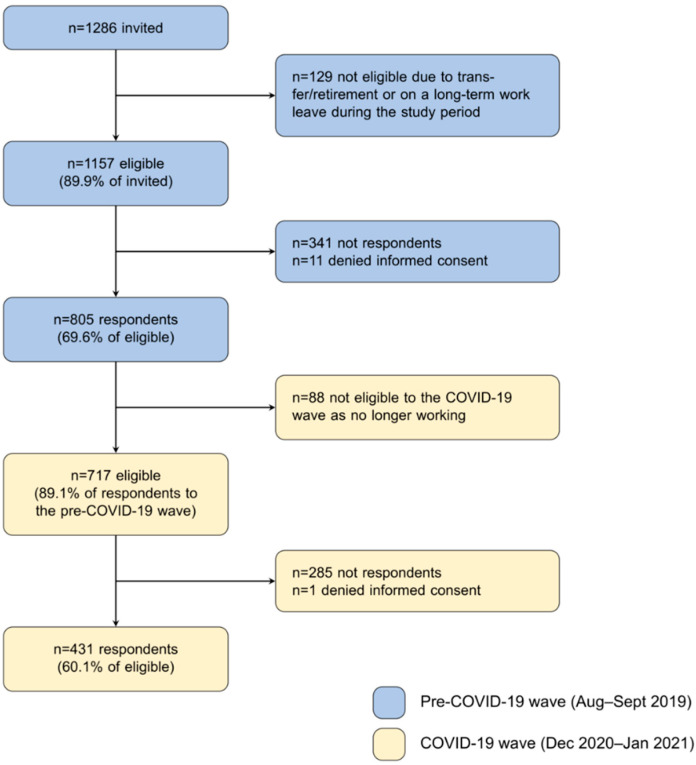
Study flow chart: invited, eligible and respondents to pre-COVID-19 (blue boxes) and to COVID-19 (yellow boxes) waves.

This is a longitudinal study carried out in a large University hospital in the city of Varese, Lombardia, the region with the highest number of SARS-CoV-2 cases and deaths in Italy [24]. The study flow chart is showed in Figure 1. N = 1286 healthcare workers were invited to participate from August to September 2019 to a periodic screening of work stress conditions required by the Italian safety legislation (from now on: pre-COVID-19 wave). These HCWs comprised all physicians, nurses, nurse assistants and front-office clerks either working in hospital wards/offices at expected high levels of work stress conditions according to representatives of the unions and of the hospital administration, or in a random sample of the remaining wards. Overall, the selected wards/offices were 67 from the following aggregated areas: emergency department and ICU, medical wards, surgery wards, front-office administration. HCWs received invitation in a sealed envelope distributed by their hospital ward referent. n = 129 invited workers who have been transferred to another ward or were on a long-term sick leave during the study period, were considered as not eligible. In December 2020, the n = 717 HCWs who responded to the pre-COVID-19 wave were still working were further invited to participate to a survey on the pandemic impact (from now on: COVID-19 wave). E-mail invitations were sent to their institutional account as required by the COVID-19 containment measures on place at that time. Questionnaires at both waves were hosted in a dedicated web site, external to the hospital organization. Participants received anonymized access credentials and were forced to change the password at first access. In addition, they were required to provide an informed consent at the beginning of the questionnaire. The study received ethical approval by the relevant ethical committee (approval ID 69/2020).

### 2.2. Assessment of Demographic and Work-Related Characteristics in the Pre-COVID-19 Wave

For each participant we collected information on age, sex, educational attainment, job title, work seniority, type of contract (permanent or fixed-term), type of employment (full time or part time), work schedule (day-time work, shift work without night shift, shift work with night shift), and area of hospital ward/office. 

### 2.3. Assessment of Work Stress and Work Satisfaction in the Pre-COVID-19 Wave

We administered the Health and Safety Executive Management Standards Indicator Tool [18], revised and validated to the Italian context (HSE-IT; [19]). The HSE-IT was developed to measure presence of workplace stressors as perceived by workers, and hence the measurement model that underpins the instrument is formative, i.e., based on the assumption that the items cause the construct being measured. HSE-IT consists in 35 items on a 1 to 5 Likert scale assessing working conditions known to potentially cause work-related stress. The original questionnaire structure comprises seven constructs: demands, control, managers’ support, peer support, relationships, role and change [18].

Furthermore, we administered a 4-item work satisfaction scale investigating satisfaction level (1–4 Likert scale). The scale was purposefully-developed for the study, adapting two items from the Copenhagen Psychosocial Questionnaire [25] referring to work prospects and to how personal abilities are used, and adding two items on work results and salary. The underlying model for this scale is reflective, i.e., based on the assumption that a latent construct “work-satisfaction” causes the responses to the items.

### 2.4. Assessment of Burnout and Mental Health

In both the pre-COVID-19 and the COVID-19 waves, we administered the Maslach Burnout Inventory, to assess burnout [26]. In the COVID-19 wave, we employed the following questionnaires: i. General Health Questionnaire–12, a measure of mental well-being [27]; ii. PTSD Checklist for DSM-5-Short Form, to assess symptoms of post-traumatic stress disorder [28]; iii. Connor-Davidson Resilience scale, assessing resilience [29]; iv. Post-Traumatic Growth Inventory–Short Form, a self-rated questionnaire assessing positive outcomes reported by people who have experienced traumatic events [30]. We adopted the validated Italian version of all these questionnaires. The psychometric properties of these scales in our study sample are assessed and discussed in a companion paper [23].

### 2.5. Statistical Analysis

We assessed representativeness of the study sample at both waves. First, we evaluated possible differences in pre-COVID-19 work-related variables between selected vs. un-selected wards/offices by comparing the following metrics: turn-over rate, up-down sizing rate, overload, short-term sick leave spell, average number of night shifts, and prevalence of regular night shifts (i.e., followed by a daily rest). These metrics were obtained starting from the hospital administrative datasets according to an established method [31] and were referred to the latest available period prior to the pre-COVID-19 wave enrollment (January 2018 to April 2019). Due to their skewed distributions, for each metric we reported the median value with 25°–75° percentiles among selected and non-selected wards/offices, and tested the null hypothesis of homogeneity of metrics using non-parametric Wilcoxon tests. A detailed analysis by job titles is reported in the Supplementary Material. Second, we investigated the amount of missing data and the presence of missing data patterns in the HSE-IT and the work satisfaction scales administered at the pre-COVID-19 wave using absolute and relative frequencies and graphical methods, respectively. The Little’s MCAR test was performed once to assess if the missing data mechanism in the HSE and in the work satisfaction scale was Missing Completely At Random [32]. Then, we explored which socio-demographic and work-related characteristics (n = 9 variables) were associated with missingness in each item of the HSE-IT (n = 35 item) and work satisfaction scale (n = 4 item) by performing chi-square analyses or Mann-Whitney tests, as appropriate, between missingness in each item and each explanatory variable. We applied the Bonferroni correction to retain statistical significance in order to control for multiple testing (corrected alpha = 0.05/((35 + 4) × 9) = 0.0007). Finally, we evaluated sample representativeness at the COVID-19 wave by contrasting socio-demographic and work-related characteristics between respondents (n = 431) and not respondents (n = 286) at the COVID-19 wave. 

We then focused on the structural validity of the questionnaires assessing pre-COVID-19 work stress and satisfaction. Since the HSE-IT is formative, the assumptions for Exploratory (EFA) or Confirmatory (CFA) Factor Analyses, as well as for Cronbach’s alpha, to assess its structure and internal consistency, respectively, are not met [33,34]. Therefore, we performed a Principal Component Analysis (PCA) to identify the components explaining the maximum variance of the HSE-IT items. Firstly, the components solutions obtained by extracting the components with eigenvalues > 1 and by examining the scree plot were compared. Since none of these methods produced a plausible components solution, we checked the components solution resulting from fixing the number of components to extract to 7, in accordance with the number of dimensions originally proposed by Cousins et al. [18]. All these solutions were interpreted after performing an Oblimin rotation to the components weights matrix. Then, as recommended for formative scales, we checked the presence of multicollinearity between the items. For each component, we performed a multiple regression in which the component was included as a dependent variable and the items that compose it as predictors, and we checked the tolerance of each item. Items whose tolerance was ≤0.35 were considered as being multicollinear.

Conversely, the factorial structure of the work satisfaction scale was assessed by performing a CFA. The fit of a unidimensional model was checked. Since the items of this scale are ordinal, a Diagonally Weighted Least Squares estimator was chosen. The fit indices that were used to evaluate the fit of the model were the Standardized Root Mean Square Residual (SRMR) and the Adjusted Goodness of Fit Index (AGFI), using the following cut-offs for model retention: SRMS < 0.08, AGFI > 0.90 [35]. Then, we assessed the internal consistency of the scale using Cronbach’s alpha, with a cutoff for the identification of adequate internal consistency at 0.70. The analyses were performed using SAS OnDemand for Academics software (release 9.04) (SAS Institute Inc., Cary, NC, USA).

## 3. Results

**Figure 2:**
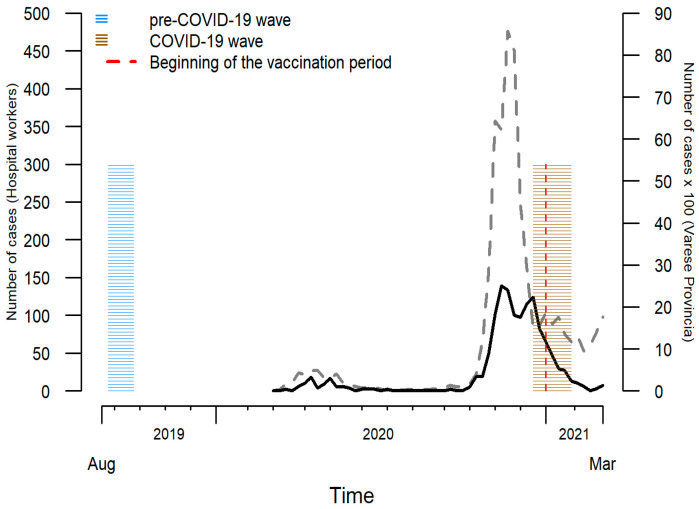
Timing of the study waves, and time trend in the weekly number of COVID-19 cases among health care workers in the investigated Varese city hospital (solid black line) and in the population living in the Varese Province (dashed grey line; cases per 100).

The timing of the study waves in relation to the pandemic curve is depicted in Figure 2. In the investigated hospital population as well as in the entire Province of Varese, the major spread of the SARS-CoV-2 pandemic started in October 2020. The COVID-19 wave took place during a downward trend in the weekly number of cases in the Varese Province, due to local mobility restriction periods. With the start of vaccination in January 2021 the epidemic reduces promptly among HCWs who were the first high-risk group involved. 

### 3.1. Sample Representativeness at the Pre-COVID-19 Wave

The n = 67 selected hospital wards and offices invited to the pre-COVID-19 wave were not different from other wards/offices with respect to the considered work-related metrics (Table 1). Further breakdown by job title (Supplementary Table S1) revealed some differences, with selected wards showing higher median values of turn-over rates among nurse assistants and administrative clerks; higher overload working hours among nurses and nurse assistants; and higher spell of short-term sick leave, but also higher regularity in night shifts scheduling, among physicians.

Participation rate at the pre-COVID-19 wave was 69.6% of eligible (805/1157; Figure 1), and more in details 54.7%, 72.3%, 67.7% and 82.9% among physicians (104/190 eligible), nurses (429/593), nurse assistants (170/251) and clerks (102/123), respectively. 

Among the 805 filled-in questionnaires, missing data was present in only 5% of the responses to the HSE-IT, and in 1.4% of the responses to the work satisfaction scale. No missing data pattern was detected by visual inspection of plots. The Little’s MCAR test was significant (χ^2^ = 1750.7, df = 1601, *p* = 0.005). However, no variable was associated with missingness in each of the HSE-IT and Work Satisfaction Scale items at the Bonferroni-corrected alpha (data not shown). Due to the low amount of missing data and the absence of variables associated with missingness in each of the items, observations with missing values were deleted from structure validity assessment.

### 3.2. Sample Representativeness at the COVID-19 Wave

Participation rate at the COVID-19 wave was 60.1% of eligible (431/717; Figure 1), and more in details 48.8%, 65.4%, 59.9% and 48.9% among physicians (42/86 eligible), nurses (261/399), nurse assistants (85/142) and clerks (43/88), respectively. Table 2 reports the socio-demographic and work-related characteristics at the pre-COVID-19 wave, among respondents and not respondents to the COVID-19 wave. We found no differences in mean age, gender distribution, work seniority, type of employment and work schedule (all *p*-values > 0.05). Conversely, participants were less likely to work in administration offices (8.6% vs. 15.7%; chi-square test *p*-value = 0.003), probably reflecting the slightly lower participation rate observed among office clerks. 

### 3.3. Structural Validity of the HSE-IT Questionnaire

Eight components had eigenvalue >1 and were firstly extracted, but the corresponding component solution (explained variance: 61%) was not plausible since a component had no weights >0.35 and another component was associated only with two items, whose weights were one positive and one negative. The analysis of the scree plot suggested that six components could better represent the data. However, the corresponding component structure (explained variance: 55%) was not plausible, since the content of the items in two components were heterogeneous and another component was the combination of two items with positive loadings and two items with negative loadings. We then extracted 7 components and the corresponding component solution (explained variance: 58%) was deemed as plausible. Table 3 reports the standardized loadings of the final component solution. 

Four of the identified components, namely demands, peer support, role and relationships, were identical to the original HSE questionnaire (Supplementary Table S2). We identified a new component comprising three items of the original change factor (“I have sufficient opportunities to question managers about change at work”, “Staff are always consulted about change at work” “When changes are made at work, I am clear how they will work out in practice”), one item from control (“working time can be flexible”) and one item from the managers’ support (“I am supported through emotionally demanding work”) factors. The items in this new component are bonded by the possibility to an active participation to shaping the changes in the work organization and to the organization of the own work. Therefore, this component was named “participation in work organization”. No item multicollinearity was apparent since the tolerance values of all items were >0.35 (data not shown).

### 3.4. Structural Validity of the Work Satisfaction Scale

The CFA on the work satisfaction scale showed that the unidimensional model had good fit (SRMR = 0.017, AGFI = 0.999). The four standardized loading ranged between 0.48 and 0.78. The Cronbach’s alpha value of 0.73 suggests adequate internal consistency. 

## 4. Discussion

Most of research on the impact of COVID-19 on the mental health of health care workers relies on cross-sectional surveys conducted during the pandemic [5,6,7,8,9,10,11,12]. As such, they cannot consider pre-pandemic levels of the investigated mental health conditions, nor their determinants such as stressful working conditions [14,16,17]. In addition, there is scanty attention to methodological issues including sample selection [5] and measurement error due to poor psychometric proprieties of the adopted scales [13], resulting in counterintuitive associations [12]. Therefore, despite their aim, any causal claim coming from existing literature should not be taken as granted.

We designed a longitudinal study with repeated measures on the same health care workers taken prior to and during the COVID-19 pandemic. To our knowledge, only two studies have a similar design. However, in one of these studies a very small sample of HCWs responded to both pre- and post-pandemic waves (n = 15; [36]), while the second on n = 153 Intensive Care Unit professionals did not measure work-related stress in the pre-COVID-19 wave [37]. With respect to these studies, we sensibly expanded the sample size and generalized the sample to a number of heterogeneous hospital wards and settings. In this paper, we focused on potential selection bias from a number of different perspectives, including the characteristics of the hospital wards from which the sample was originated, participation rates at both study waves, and data completeness. Taken together, our findings consistently point towards a satisfactory representativeness of the study sample with respect to the operating health care workforce population.

From a methodological viewpoint, the adoption of the HSE and its Italian version to investigate perceived work stress may suffer from two major drawbacks: the use of inappropriate analyses to validate its psychometric validity, and the lack of a formal validation in the specific HCWs population. The study which developed the HSE [18] and the studies that assessed its structural validity employed EFAs or CFAs and Cronbach’s alphas [19,20,21,22], which are inappropriate if there is no latent factor causing the responses to the questionnaire, as in formative scales. The improper use of EFAs and CFAs may have important consequences, such as inadequate selection of items during the development of the scale, misrepresentation of constructs, biased estimates of the relationship between the item and the latent variable or of the relationship between the latent variable and other variables [33,34]. Regarding the Italian version, Magnavita [20] on a sample of 748 workers from 17 companies employed a Principal Component Analysis, as appropriate for the assessment of formative constructs, but interpreting results as if it were an EFA. Hence, he suggests the use of the identified structure only for the Italian version, not noting that the scale was previously analyzed as if it was reflective. Therefore, we suggest that other studies should address the structural validity of the HSE using the appropriate statistical methods.

The PCA allowed us to find a 7-component solution explaining 58% of the variance whose components had a plausible content. Similarly to the PCA performed by Magnavita [20], in both cases 7 components were extracted and the content of five of them overlap. In contrast, Magnavita did not interpret the 7th component since no significant loadings were present; and identified an “Elasticity” component which corresponds to the sum of the “Managers’ support” and the “Participation to the work organization” components found in our study. Employee participation is a key concept for the understanding of the relationship between the worker and the work context, besides being known as a determinant of organization performance [38]. Therefore, our newly identified component has a sound theoretical background. Similarly to the study by Magnavita, item 33 (“I am supported through emotionally demanding work”) cross-loaded on two components, namely “Participation to Work Organization” and “Peer support”. Rather than to the influence of the specific organizational setting, this might be due to the content of the item, which can be interpreted by the respondent as receiving either formal or informal support from multiple, unspecified sources. However, since ours is the first attempt to validate HSE-IT among HCWs, the removal of the item requires future confirmation in other studies. Until then, we recommend using our component structure to assess work stressors in HCWs samples.

We acknowledge the following limitations. First, our longitudinal design has only one assessment during the COVID-19 pandemic, and therefore it is unable to investigate whether the effects on mental health are short- or long-term ones. At the time we are writing this paper, the pandemic has not ended yet. Second, we did not administer HSE-IT at the COVID-19 wave. Therefore, we are not able to assess whether the 7 components’ structure observed in our study is time-invariant with respect to the pandemic outbreak. In addition, we will not be able to evaluate changes in perceived work stress and work satisfaction due to the COVID-19 pandemic. Finally, our study involved only one hospital in Northern Italy, and hence future research is desirable to confirm and generalize the structural validity of HSE-IT and work satisfaction in different healthcare settings. Among the study strengths, we mention: the longitudinal study design with a representative and large sample at both pre-COVID-19 and COVID-19 waves; the adoption of several mental health outcome scales; and the assessment of protective factors which help individuals to maintain the psychological wellbeing, such as resilience [29] and post-traumatic growth [30]. A parallel paper addresses the structural validity of the outcome scales, including measurement invariance over time, as well as of their potential mediators and effect modifiers [23]. Taken together, the two companion papers can provide useful suggestions on methodological issues related to the causal link between pandemic and mental health. 

In conclusion, our study is based on a representative sample of HCWs from several hospital wards. The identified 7-component structure for HSE-IT, including a new “participation in work organization” component, explained a large amount of variance of perceived work stress, and it can be adopted in the health care workers population. Due to its longitudinal design, our study is well-suited to elucidate the role of work conditions, registered before the pandemic, on the development of mental health disorders among health care workers.

## Figures and Tables

**Table 1:** Assessing representativeness of the surveyed sample: medians and interquartile ranges (25th–75th percentiles) of turn-over, up/down sizing, overload, spell of short-term sick leaves, number of night shifts and night shifts regularity in recruited and non-recruited wards.

Metric	Unit of Measurement	Recruited Hospital Wards/Offices (n = 67)	Other Hospital Wards/Offices (n = 297)	*p*-Value ^
Turn-over	number per 100 HCWs	38.9 (15.8; 68.3)	28.9 (0.0; 63.6)	0.11
Up/down sizing	number per 100 HCWs	0.0 (−8.9; 4.8)	0.0 (−9.3; 0.0)	0.44
Overload	hours/week per HCW	1.5 (0.6; 2.4)	1.1 (0.4; 2.2)	0.11
Spell of short-term * sick leave	number per 100 HCWs	124.5 (62.6; 175.2)	119.7 (42.0; 205.1)	0.75
Night shifts	number per HCW	58.5 (20.5; 75.9)	47.9 (14.4; 79.2)	0.56
Regular night shifts	percent	85.3 (41.4; 96.6)	65.5 (32.9; 97.5)	0.54

In the table: median (25th–75th percentile). Abbreviations: HCWs = health care workers Metrics are referred to the period: 01 January 2018–30 April 2019 (prior to the pre-COVID-19 wave). *: <3 days. ^: Wilcoxon rank test.

**Table 2:** Socio-demographic and work-related characteristics assessed at the pre-COVID-19 wave, for respondents and not respondents at the COVID-19 wave.

	Respondents (n = 431)	Not Respondents (n = 286)	*p*-Value ^
**Age, years**	46.5 ± 9.5	46.5 ± 10.0	0.94
**Men, n (%)**	83 (19.3%)	70 (24.1%)	0.12
**Educational attainment, n (%)**			
Less than high school	50 (11.6%)	42 (14.7%)	0.16
High school	185 (43.0%)	104 (36.4%)	
University degree	195 (45.4%)	140 (49.0%)	
**Work seniority, years**	16.2 ± 10.9	15.1 ± 11.4	0.21
**Type of employment, n (%)**			
Full-time	364 (85.7%)	250 (87.7%)	0.25
Part-time	66 (15.3%)	35 (12.3%)	
**Work schedule, n (%)**			
Day-time work	69 (16.1%)	60 (21.1%)	0.18
Shift work w/o night shift	83 (19.3%)	46 (16.1%)	
Shift work with night shift	278 (64.6%)	179 (62.8%)	
**Hospital ward/office, n (%)**			
Emergency Department	182 (42.2%)	120 (42.0%)	0.003
Medical wards	80 (18.6%)	60 (21.0%)	
Surgical wards	132 (30.6%)	61 (21.3%)	
Administration offices	37 (8.6%)	45 (15.7%)	

In the table: mean ± standard deviation for continuous variables, and n (%) for categorical variables. ^: *p*-value comparing respondents to not respondents, from *t*-test for continuous variables and chi-square test for categorical variables.

**Table 3:** Loadings of the HSE items on the components identified through Principal Component Analysis.

Item	Demands	Control	Peer Support	Manager’s Support	Role	Participation in Work Organization	Relationships
Item 3	**0.64**	−0.06	0.06	0.03	0.22	0	−0.06
Item 6	**0.69**	−0.03	0.07	0.04	0.19	−0.11	0.05
Item 9	**0.62**	0.14	−0.04	−0.04	−0.28	0.07	0.11
Item 12	**0.69**	−0.07	0	−0.08	0.05	0.20	0.07
Item 16	**0.42**	0.21	−0.12	0.16	−0.16	0.01	0.15
Item 18	**0.34**	−0.03	−0.03	0.28	−0.03	0.02	0.23
Item 20	**0.57**	0.20	−0.03	−0.01	−0.28	0.06	0.12
Item 22	**0.73**	0.03	0.11	0.02	0.09	−0.04	0.01
Item 2	0.24	**0.60**	0	0.04	−0.14	−0.03	−0.06
Item 10	0.06	**0.80**	0.04	−0.05	0.09	0.03	−0.02
Item 15	−0.08	**0.80**	−0.02	0.07	0.14	−0.02	0.03
Item 19	−0.13	**0.77**	0.10	0.05	0.03	0.08	−0.05
Item 25	−0.09	**0.59**	0.13	0.03	0.19	0.18	0.20
Item 7	0.14	0.07	**0.73**	0.19	−0.07	−0.21	−0.09
Item 24	0.03	0.10	**0.78**	0.08	−0.07	−0.02	0.10
Item 27	−0.11	0.13	**0.62**	−0.02	0.13	0.01	0.28
Item 31	0	0.02	**0.80**	−0.06	−0.07	0.16	0.04
Item 8	0.15	0.10	0.35	**0.41**	0.03	0.03	−0.22
Item 23	0	0.04	0	**0.87**	0.03	−0.01	0.03
Item 29	−0.09	0.04	0.02	**0.79**	−0.01	0.05	0.17
Item 35	−0.04	0.02	0.02	**0.83**	0.03	0.10	−0.03
Item 1	0.17	0.07	0.17	−0.04	**0.62**	0.13	−0.08
Item 4	−0.03	0.12	−0.11	−0.07	**0.60**	−0.10	0.12
Item 11	0	0.08	−0.02	0.12	**0.63**	−0.11	0.18
Item 13	0.07	−0.04	0.03	0.24	**0.61**	0.24	−0.07
Item 17	0.16	−0.06	0	0.09	**0.56**	0.37	−0.13
Item 26	−0.03	0.12	0.05	0.3	0.01	**0.53**	0.09
Item 28	−0.04	0.01	0.10	0.22	−0.02	**0.64**	0.10
Item 30	0.05	0.15	−0.10	0	−0.15	**0.59**	0.01
Item 32	0.05	−0.03	0.05	−0.01	0.23	**0.68**	−0.03
Item 33	0.06	0.13	0.35	0.15	0.01	**0.39**	−0.09
Item 5	0.18	0.07	−0.03	0.10	0.02	−0.03	**0.67**
Item 14	−0.04	−0.1	0.46	−0.05	0.07	0.18	**0.53**
Item 21	0.14	0.06	−0.01	0.17	0.02	0.04	**0.68**
Item 34	0.07	0	0.23	0	0.11	−0.02	**0.48**

Note. Adjacent loadings belonging to the same component are marked in bold.

## Data Availability

Anonymized study data are available upon motivated and reasonable request to the corresponding author.

## Supplementary Materials

The following supporting information can be downloaded at: https://www.mdpi.com/article/10.3390/ijerph19159514/s1, Table S1: Assessing representativeness of the surveyed sample, by job title: medians and interquartile ranges (25th–75th percentiles) of turn-over, up/down sizing, overload, spell of short-term sick leaves, number of night shifts and night shifts regularity in recruited and non-recruited wards; Table S2: Comparison between the results of the Principal Component Analysis and the results of the analyses performed by previous studies on the structural validity of the HSE.
