# Supplementary material for: The North Italian Longitudinal Study Assessing the Mental Health Effects of SARS-CoV-2 Pandemic on Health Care Workers—Part I: Study Design and Psychometric Structural Validity of the HSE Indicator Tool and Work Satisfaction Scale
Source: Int J Environ Res Public Health. 2022 Aug 3;19(15):9514. doi: 10.3390/ijerph19159514 (PMC9368475; doi:10.3390/ijerph19159514)
Supplement: Supplementary file 1 [file ijerph-19-09514-s001.zip › ijerph-1804514-supplementary.pdf]

The North Italian Longitudinal Study assessing the Mental Health effects of SARS-CoV-2 pandemic on Health Care Workers - Part I: study design and psychometric structural validity of the HSE Indicator Tool and work satisfaction scale

Giovanni Veronesi, Emanuele Maria Giusti, Alessia D'Amato, Francesco Gianfagna, Rossana Borchini, Gianluca Castelnuovo, Licia Iacoviello, and Marco Mario Ferrario

Supplementary Tables

Table S1. Assessing representativeness of the surveyed sample, by job title: medians and interquartile ranges (25th-75th percentiles) of turn-over, up/down sizing, overload, spell of short-term sick leaves, number of night shifts and night shifts regularity in recruited and non-recruited wards

Job title	No. of wards	Turn-over	Up/down sizing	Overload	Spell of short-term* sick leave	Night shifts	Regular night shifts
**Physicians**							
Recruited hospital wards	30	31.8 (0.0; 61.9)	0.0 (-9.9; 7.5)	3.0 (1.9; 4.1)	62.6 (42.8; 88.1)	32.3 (16.0; 48.0)	39.9 (28.5; 47.4)
Other hospital wards	112	19.1 (0.0; 57.0)	0.0 (0.0; 9.6)	2.4 (0.8; 4.5)	34.0 (0.0; 102.2)	16.2 (6.2; 40.1)	30.6 (7.6; 38.2)
*p-value^*	-	0.33	0.76	0.47	0.03	0.07	0.01
**Nurses**							
Recruited hospital wards	38	44.1 (18.9; 69.5)	-1.7 (-10.9; 0.0)	1.7 (1.0; 2.2)	120.7 (87.7; 155.5)	79.9 (68.3; 85.5)	96.7 (95.4; 98.2)
Other hospital wards	166	34.2 (0.0; 78.5)	0.0 (-8.8; 0.0)	0.6 (0.3; 1.5)	151.7 (72.5; 240.0)	81.9 (65.8; 92.9)	98.0 (94.7; 99.5)
*p-value^*	-	0.37	0.26	0.0003	0.18	0.20	0.16
**Nurse assistants**							
Recruited hospital wards	36	70.4 (22.8; 132.6)	0.0 (0.0; 21.1)	1.0 (0.5; 1.3)	183.1 (93.1; 281.6)	71.9 (15.6; 90.3)	97.0 (92.8; 98.5)
Other hospital wards	116	26.7 (0.0; 102.3)	0.0 (0.0; 0.0)	0.3 (0.0; 1.0)	196.3 (90.3; 308.9)	77.9 (23.5; 93.5)	98.7 (97.2; 99.8)
*p-value^*	-	0.03	0.05	0.001	0.92	0.39	0.11
**Administrative workers**							
Recruited hospital wards	20	25.2 (0.0; 74.1)	0.0 (0.0; 0.0)	0.4 (0.2; 0.8)	171.2 (118.4; 231.5)	nr	nr
Other hospital wards	101	0.0 (0.0; 49.3)	0.0 (0.0; 0.0)	0.5 (0.2; 1.2)	119.5 (0.0; 225.6)	nr	nr
*p-value^*	-	0.04	0.37	0.34	0.08	-	-

In the table: median (25th-75th percentile).
Turn-over: number per 100 HCWs. Up/down sizing: number per 100 HCWs; Overload: hours/week per HCW; Spell of short-term sick leave: number per 100 HCWs
Night shifts: number per HCW; Regular night shifts: percent
Metrics are referred to the period: 01/01/2018-30/04/2019 (prior to the pre-COVID-19 wave)
*: < 3 days
^: Wilcoxon rank test

Table S2: Comparison between the results of the Principal Component Analysis and the results of the analyses performed by previous studies on the structural validity of the HSE

		Component in which the item loaded		
Item #	Item content	Cousins et al., 2004	Magnavita et al., 2012	This study
Item 3	Different groups at work demand things from me that are hard to combine	Demands	Demands	Demands
Item 6	I have unachievable deadlines	Demands	Demands	Demands
Item 9	I have to work very intensively	Demands	Demands	Demands
Item 12	I have to neglect some tasks because I have too much to do	Demands	Demands	Demands
Item 16	I am unable to take sufficient breaks	Demands	Demands	Demands
Item 18	I am pressured to work long hours	Demands	Demands	Demands
Item 20	I have to work very fast	Demands	Demands	Demands
Item 22	I have unrealistic time pressures	Demands	Demands	Demands
Item 2	I can decide when to take a break	Control	Control	Control
Item 10	I have a say in my own work speed	Control	Control	Control
Item 15	I have a choice in deciding how I do my work	Control	Control	Control
Item 19	I have a choice in deciding what I do at work	Control	Control	Control
Item 25	I have some say over the way I work	Control	Control	Control
Item 7	If work gets difficult, my colleagues will help me	Peer support	Support	Peer support
Item 24	I get help and support I need from colleagues	Peer support	Support	Peer support
Item 27	I receive the respect at work I deserve from my colleagues	Peer support	Support	Peer support
Item 31	My colleagues are willing to listen to my work-related problems	Peer support	Support	Peer support
Item 8	I am given supportive feedback on the work I do	Managers' support	Elasticity	Managers' support
Item 23	I can rely on my line manager to help me out with a work problem	Managers' support	Elasticity	Managers' support
Item 29	I can talk to my line manager about something that has upset or annoyed me about work	Managers' support	Elasticity	Managers' support
Item 35	My line manager encourages me at work	Managers' support	Elasticity	Managers' support
Item 1	I am clear what is expected of me at work	Role	Role	Role
Item 4	I know how to go about getting my job done	Role	Role	Role

Item 11	I am clear what my duties and responsibilities are	Role	Role	Role
Item 13	I am clear about the goals and objectives for my department	Role	Role	Role
Item 17	I understand how my work fits into the overall aim of the organisation	Role	Role	Role
Item 26	I have sufficient opportunities to question managers about change at work	Change	Elasticity	Participation in work organization
Item 28	Staff are always consulted about change at work	Change	Elasticity	Participation in work organization
Item 30	My working time can be flexible	Control	Elasticity	Participation in work organization
Item 32	When changes are made at work, I am clear how they will work out in practice	Change	Elasticity	Participation in work organization
Item 33	I am supported through emotionally demanding work	Managers' support	Elasticity	Participation in work organization
Item 5	I am subject to personal harassment in the form of unkind words or behaviour	Relationships	Relationships	Relationships
Item 14	There is friction or anger between colleagues	Relationships	Relationships	Relationships
Item 21	I am subject to bullying at work	Relationships	Relationships	Relationships
Item 34	Relationships at work are strained	Relationships	Relationships	Relationships
